# Verdict against an Apothecary for an Alleged Mistake in Putting up a Prescription

**Published:** 1866-11

**Authors:** J. R. Lothrop


BUFFALO

### Medical and Surgical Journal.

VOL. VI. NOVEMBER, 1866. No. 4.

### Original Communications.

ART. I.—*Verdict against an Apothecary for an alleged mistake in putting up a Prescription.* By J. R. Lothrop, M. D.

(CONCLUDED.)

In a preceding article an account was given of testimony in the case, some of which was certainly of a most extraordinary character. In the present, there will be an attempt to state what is at present known or believed, upon the subject of the action and properties of veratrum viride. At the trial it was stated by the main medical witness for the plaintiff, that all the hellebores contained the alkaloid veratria. The kinds of hellebore were enumerated, viz: the white, green and black. It is needless to state to a well informed medical man, that black hellebore has no botanical affinity with the others, is not classed with the others in any system of Materia Medica, and has never been stated to contain the alkaloid veratria. Of course it will be perceived, the bearing of a question on the part of counsel to bring out such a statement, was to make a strong impression upon the minds of the jury, as to the poisonous nature of hellebore by whatever name known, whether white, green, or black. It was established that veratria was a poison, and if all hellebores contained veratria, they were poison also. Unfortunately on the part of the defence the error of this statement was not clearly brought out, so that with the confusion of several kinds of hellebore, and that arising from the names veratrum and veratria, it would not be strange if the jury were convinced that all hellebores contained veratria and were equally poisonous. As regards veratria, it is true, it is an intestinal irritant, and will produce bloody discharges for a longer or shorter time, and is said by some to cause intestinal inflammation; this is equally, or we may say more true, of the veratrum album from which it is obtained.

It is, then, an important inquiry: does veratrum viride or green hellebore, contain the alkaloid veratria? Dr. Osgood, who wrote an elaborate essay upon the properties and actions of veratrum viride, stated that he obtained by chemical process a white powder which he supposed to be the alkaloid of the plant. Mr. Thos. A. Mitchell also obtained by analysis a white powder which he thought to be the active principle. But neither of them declared, if they suspected, that the white powder found by them resembled in any way veratria. In fact if they did make any chemical experiments to prove its identity with veratria, they did not announce them, and Dr. Osgood only states that the powder was inodorous and acrid.

Not long after Mr. Worthington made a complete analysis, and the result of his researches was the finding of "an alkaloid substance identical with veratria." This was the first announcement that the plant contained such an alkaloid. Not long since Mr. Richardson satisfied himself by careful analysis and tests, that an alkaloid identical with veratria existed in the veratrum album. About the same time Prof. Percy of New York, obtained from the plant "a very fine, white, amorphous powder, possessing all the physical properties of veratria." In 1862 Mr. Scattergood of Philadelphia made a very careful comparison of the alkaloids derived from the white and green hellebore, and he also was satisfied that they were identical.

On the other hand, Mr. Charles Bullock of Philadelphia, in two recent numbers of the *Journal of Pharmacy*, Sept. 1865 and March, 1866, after giving the processes employed by him to separate, and the re-actions of the active principle, (or principles, as he found two,) of veratrum viride, makes this statement: "The re-actions of both these principles with sulphuric acid, carefully repeated, and with hydrochloric acid, tend to the belief that neither of them is veratria, properly considered, although this and the deportment with other re-agents, show a close connexion between them." In a second communication in March, 1866, after stating that the object of his second investigation was "to obtain information whether the alkaloids obtained from the plant (veratrum viride) were *identical* with veratria," he goes on to say as one point in the summary of his investigation, "that veratrum viride contains two alkaloids—one soluble in ether, and the other insoluble in that menstruum. *Neither of these principles answer in their chemical re-actions to veratria.*"

Prof. Percy states, that "sulphuric acid is the only one of all these [re-agents] that can be deemed decisively confirmatory of the presence of veratria." With this acid, veratria changes from a light yellow to bright blood red, then crimson, which latter color lasts some hours. According to Mr. Bullock the alkaloids of veratrum viride when treated with concentrated sulphuric acid give the following re-actions: "dissolving to a reddish yellow color, which changes to ochery red, then to reddish brown, and finally becoming brown." This re-action, if reliable, differs essentially from that of veratria with the same re-agent.

It was shown by Trapp of Russia in 1863, that veratria in cold hydrochloric acid dissolved to a *colorless* solution; when boiled it assumed a red color, that finally became intense, resembling that of permanganate of potassa. Of this test Mr. Bullock remarks: "this re-action is very sensitive, even with impure veratria of commerce—the color remaining with little change in a closed test tube, after the lapse of ten days." He found, however, upon applying this test to the alkaloids of veratrum viride that both "dissolved in cold hydrochloric acid to faint *yellow* solutions; boiling deepened this color. After standing twenty-four hours, the solutions assumed a turbid *greenish* color." This re-action again, it will be perceived is very unlike that of veratria with the same re-agent.

These re-actions have been detailed to show that the investigations of Mr. Bullock are the most recent, and apparently made with great care; and they seem to be very decisive in their bearing upon the question of the existence of an alkaloid in veratrum viride identical with veratria. They certainly show a very great dissimilarity in their chemical properties. They are not therefore identical. We may assume that their therapeutical actions are also different, though this is rather a matter for proof than inference, as substances having like chemical affinities do not always have like actions as medicines.

But apart from inference, the observations of Prof. Percy and others clearly indicate that the therapeutical action of the alkaloids derived from veratrum viride is not identical with that of veratria, and though the difference in the action of the alkaloids is not so great as that in the actions of the plants from which they are obtained, viz: the veratrum viride and album; yet it is distinct enough to warrant the belief that they are not the same.

Dr. Stillé in his excellent work says, "the botanical affinities of the plant, and its action upon the animal economy, render it probable that its virtues are owing to *veratria*, the active principle of *veratrum album*." In his first edition he added, "but the experimental proof of this supposition is still wanting." In the second edition this last clause is omitted, though he gives nothing new which seems to have determined him to give over his doubts and admit the proposition, except a statement that Dr. Uhle of Dorpat, analyzed a specimen of veratria obtained from the green hellebore sent him from New York, and he found it identical with that obtained from the veratrum album.

Dr. Wood in the latest edition of the Dispensatory, after noticing the most recent investigations as to the properties and actions of the veratrum viride, says: "A very important inference is that there is a principle in the American hellebore distinct from veratria, upon which its remarkable powers over the circulation depend."

To sum up the authorities upon the subject, if we give most weight to the latest investigations, we are warranted in saying that, as far as the chemical properties are concerned, the alkaloids derived from veratrum viride and veratrum album are entirely distinct, and that veratrum viride does not contain veratria. It must, however, be conceded that the opposite opinion is supported by the greater number. We must on all questions weigh authorities as well as count them.

When we consider the question in its therapeutical aspect, we are clearly entitled to infer that no identity of action exists, or has been claimed. The alkaloids from both plants are stated to act similarly, but not exactly alike. But even admitting that the alkaloids from both plants are identical in properties and actions, Prof. Percy states that the alkaloid exists in the veratrum viride in very small quantity, so that in a considerable dose the amount would be too small to expect from it any sensible effect.

When we consider the actions of the plants themselves, we find they differ widely in their effects. This of itself leads to the inference that the active principles of the plants are different. If we admit that the alkaloids of each when isolated act pretty much alike, we shall be obliged to assume that in the *whole* plant their action is modified by other principles that account for the difference. In the case of veratrum viride it is something distinct from veratria, as we get it from veratrum album. But in the case of veratria from the veratrum album, its effects may be taken to be, in the main, those of the whole plant from which it is obtained. We must then, conclude, either that the alkaloid isolated from veratrum viride and called veratria, is not the same as that obtained from the veratrum album; or that if the same, it is in the plant in such small quantity, or so associated with other principles, which are more active, that it loses its power to act at all as veratria. In either case it is not to be taken into the account, in estimating the actions of veratrum viride; for either it is not veratria, or if veratria, it loses its distinct action by association with other principles. Veratria obtained from the veratrum album, is spoken of by Dr. Stillé as causing in animals "a copious discharge of liquid with mucus and even blood from the bowels, with spasmodic retraction of the abdominal walls;" if veratrum viride contains the same alkaloid, what is it that robs it of its power, when in the plant, to produce these effects?

This brings us to the question of so much importance in the case above related, viz: whether veratrum viride can in any proper sense be called a purgative. It is well known that one of the marked effects of veratrum album, when given in full doses, is excessive purging; frequent discharges of mucus and blood taking place. It is undeniably in large doses a violent irritant of the bowels, pretty uniformly causing purgation. In this respect it differs radically from veratrum viride, which, according to the experience of most observers, never, or very rarely acts as a purgative. This difference is so radical as to constitute in itself a sort of proof that the active principle in each is different.

If we examine the testimony of writers upon the subject we find this statement confirmed by the great majority. Dr. Osgood, in his essay in 1835, said, after a pretty free use of it, that according to his experience the veratrum viride "has not the slightest laxative effect." Much earlier than this, Dr. John Ware of Boston, gave it in thirty cases, in order to ascertain its effect upon the stomach and alimentary canal. He used the powder in doses as large as ten grains, and "in no instance was it very clear that purging was produced." Dr. Norwood, well known in connection with this plant, stated: "it is not cathartic by any means." Mr. Worthington, in several trials upon himself, found that "in neither trial was there a disposition to catharsis." Dr. John Bell of Brooklyn, in a Report on Materia Medica, published in the *North American Medico-Chirurgical Review* for Sept. 1858, gives a summary of the experience of some sixteen practitioners, in various parts of the country, in the use of veratrum viride. Some of them used very large doses, and by none is it distinctly stated that purging occurred. Had such an action taken place it would hardly have failed of mention, on account of the serious interference with the continued use of the medicine, which such an effect would have caused. On the other hand many of them stated, as one of the advantages of the medicine, that it did not purge. Thus Dr. Boerstler of Lancaster, Ohio, reports: "I have given it to patients for two months, and never witnessed any catharthic action;" and Dr. Hutchinson states: "In no instance have I found veratrum [viride] to purge."

Prof. Percy gives the most positive testimony on this point. He says: "I have carefully watched over one thousand cases for this effect, and I have never seen a case in which I was satisfied that purging was produced by this alone, when given by the mouth."

Dr. Stillé quotes the statements of Drs. Osgood and Norwood, above given, and adds: "To these accounts most of the reporters subscribe." These "reporters" include many of the best men of the profession, among them Prof. McGugin of Iowa, whose reports are of great interest and value. But he finds that in *one* case, Dr. E. M. Pendleton reports "copious watery evacuations *per anum;*" and in two cases Dr. E. Platt reports "profuse watery evacuations from the bowels," and "looseness of the bowels." In spite of all the positive testimony on the other side, the report of these two practitioners in three cases, inclines him to believe that the denial of purgative properties to veratrum viride, is not yet to be fully accepted as a settled question.—This is to be inferred from his language; for after stating that in three cases Dr. Rayner found that veratrum album, given so as to cause dangerous symptoms, yet did not purge, he says: "It is therefore certain that if an absolute difference exists between the two medicines, it is not to be found in their cathartic operation;" although he admits that "it would appear that this effect is most usual with white hellebore." This certainly so leaves the matter of difference between green and white hellebore as to cathartic action, as to give it very little prominence, and in fact lead the reader to suppose that hardly any exists. From all the testimony which might be adduced on the subject, the matter would be much more fairly stated as follows: All writers agree in ascribing to the white hellebore a most decided action, as an intestinal irritant, hence causing excessive purging, not easily controlled. This action is not without exception, but is the rule, and it is pretty safe to state that a large dose will give rise to catharsis. On the other hand, with veratrum viride, the rule is, no intestinal irritation and no catharsis; and so constantly, that it may be fully stated to be almost without exception. This difference, if not positively absolute, is yet sufficiently distinct, to entitle it to more prominence than is given to it by Dr. Stillé.

Comparing authorities, it appears that the great majority of observers are positive in declaring that veratrum viride is not a purgative, and we seem warranted in asserting that the weight of authority is on the same side. Undeniably, in some cases, its use has been followed by free movements of the bowels, but this action without doubt, in many instances, follows from its emetic action, and often follows any emetic. The secretions are so increased by emesis, that watery evacuations may take place by the bowels; but this is temporary, and not in any true sense purging. It seems safe to assert, that no evidence can be found in the experience as to the effects of veratrum viride, as yet announced, which goes to prove that it has any positive operation as an intestinal irritant, and therefore liable to cause excessive purging, with mucus and bloody discharges.

As bearing upon this point, cases in which very large, even in some cases, excessive doses have been used are here detailed.

Dr. Hutchinson of Indiana, gave to a boy three years of age, with croup, in twenty-four hours, one ounce of the tincture of veratrum viride without producing much vomiting or any purging, and "with the happiest results." Dr. Watson states, in the *Nashville Journal*, that he gave one patient 16 drops of the tincture, every two hours, three days in succession, "without the slightest effect."

Dr. Barker, in his service at the Bellevue Hospital, in a case of puerperal fever, made use of the tincture for six successive days, giving on some days as many as 10 drops every hour for several hours. The first day, in 9 hours, 72 drops were taken; on the second, during the twenty-fours, 68 drops; on the third, 82 drops; on the fourth, 91 drops; on the fifth, 78 drops; on the sixth, 62 drops; in all 453 drops in 5½ days—equal to 5 or 6 drachms. At the end of the sixth day the report was, "feels well, improvement marked." These quantities caused some vomiting; nothing like purging; no bad effects.

Dr. Newson of Georgia, mentions three cases in which large doses were taken to procure abortion, in two, half teaspoonful doses of tincture, repeated in an hour; in one, two doses of thirty drops each, (interval not mentioned,) in all cases with no bad effect beyond extreme nausea. Dr. Taliaferro mentions the case of a physician of Atlanta, Ga., who took by mistake an ounce of the tincture at one time; the disturbance only amounted to extreme nausea and vomiting, and some difficulty in breathing. A case occurred in the practice of Dr. Loomis of this city, in which by mistake a teaspoonful of the tincture was taken at one dose. The consequences of this mistake were extreme vomiting, one or two movements of the bowels, but nothing like purging, great prostration, readily rallied from. The patient was afflicted with acute rheumatism, which was cured by the dose. Prof. Percy in his Essay gives, in a note, four cases in which enormous doses were taken, and in neither, is purging mentioned as a result, only vomiting and great prostration. These effects were but temporary.

These cases are so remarkable that it is worth while to give some of the details, for those who may not have met the account of them.

A farmer put ten pounds of green hellebore in water and boiled to a gallon; of this decoction, by mistake, his wife drank a tumbler full; free vomiting, prostration, and fearful cold sweat followed, but she recovered in forty-eight hours.

In the second, two quarts of syrup were prepared from two pounds of green hellebore, by mistake, instead of valerian. Of this syrup a tumbler full was taken, and as it caused vomiting the dose was repeated. The alarming symptoms above given followed, but in two days the recovery was complete.

A physician, by mistake, took thirty grains of the active principle [the alkaloids and resinous extract combined] of veratrum viride. It caused copious vomiting, prostration, loss of pulse at the wrist, but on the third day he was well again.

In the fourth, a man drank from a bottle marked "old rum," nearly a tumbler full of the tincture of veratrum viride; vomiting followed, yet genuine rum being freely given, he walked home the same night.

If the question of peril to life were under discussion, these cases would be very important in their bearing upon it, for they show how little real danger there is in excessive doses. But as no case of death resulting from over-doses of veratrum viride is on record, it is more in keeping with the purpose we have in hand, to remark the striking fact that no purgative effect is spoken of in either. This part of the subject will be closed with Dr. Bell's conclusion, after collecting the evidence of various physicians who had made use of the medicine. He says: "The veratrum viride differs from the veratrum album in one essential particular, viz: in its exerting little or no action on the bowels as an aperient." He thinks it might even be employed as a corrigendum, with drastic purgatives, acting in that respect very much like hyosciamus.

It will not escape observation that in all the cases related above, in which excessive doses were given, the tincture or decoction was the form used. Whereas in the case which is related in this paper,

Thayer's fluid extract was the form employed. As the question arose, of the relative strength of the fluid extract and tincture, and the counsel for the plaintiff made much of the greater strength of the extract, it is worth while to examine it somewhat. It was assumed that the tincture being a simple alcoholic solution, and the fluid extract a solution concentrated by evaporation, it is plain enough that, from the method of preparation, the latter is the stronger.

Now Norwood's tincture and that of the Dispensatory, are both meant to be saturated solutions; and it seems perfectly plain that, we cannot gain in strength beyond the capacity of the menstruum for solution. If we attempt concentration, we produce a deposit, as the portion held in solution, when deprived by evaporation of its alcohol, falls to the bottom. Unless the bottle containing the fluid extract is well shaken at the time of dispensing; or sugar is added so that a syrupy liquid is produced by evaporation, capable of suspending the portions which in a thinner liquid would fall to the bottom, it is difficult to understand how the strength can vary much in each.

Dr. Wood in the Dispensatory says, the fluid extract may be only as strong, or it may be stronger; probably taking into account the conditions above named, viz: agitation and density of the menstruum. Practically Dr. Thayer's solution is as liquid as the tincture, and usually lets fall a deposit when allowed to stand undisturbed for a time. It does not seem very reasonable to expect anything beyond saturation of a mentruum, in liquids equally dense. Such being the case we should hardly expect 10 drops of the fluid extract to exceed in strength 10 drops of the tincture. There can be no essential difference.

On the trial, it was stated by the medical witness for the plaintiff, that the pulse is reduced by veratrum viride in a marked degree, and may be brought down as low as twelve beats per minute. He was unable to state whether the plaintiff's pulse was brought down to that number. The pulse could not be felt at the wrist, and he did not listen to the sounds of the heart. Dr. McCollum did; but he was not willing to commit himself to quite so low a rate, though he could not state accurately how often the heart beat per minute. He was of the impression that it was not less than thirty.

Prof. Percy does not state distinctly that he has observed a reduction of the pulse below 30 beats per minute, but, he says, in a case related to him, it was stated that the reduction was as low as 19 per minute. No case can now be brought to mind by me, in which there is any account of a lower rate than 30 per minute, as the effect of veratrum viride. A possible reduction to 12 beats per minute may be conceded, but very few practitioners will probably be able to report it from their own observation, from any cause. In chronic diseases of the brain, the pulse is often very slow. Todd and Bowman mention a case under their observation, in which, for months, the pulse was as low as 16 per minute. Fordyce speaks of its being at 26 and 20 per minute.

The counsel for the plaintiff asked: if veratrum viride reduces the action of the heart, so that it beats very slowly, may it not, getting slower and slower, by and by stop? It would not be very strange if a jury should get an impression that, when the beating of a man's heart had got as low as 12 per minute, it was pretty near the stopping point. In fact, most medical men would feel rather uneasy counting a pulse of 12 only per minute, especially after having given veratrum viride.

It seems to me, that in reviewing the foregoing account, it may be fairly claimed that it has been shown: first, that, although without doubt, veratrum viride contains an alkaloid, which has been repeatedly isolated, it does not appear to be established that it is identical with veratria either in chemical or therapeutical actions.

Secondly, conceding that its alkaloid is identical with veratria, it is not the active principle of the plant, and hence the cause of its peculiar action; but, either, because of its association with other principles which modify its powers, or because it exists in such small quantity, it has no tendency to act in the plant as veratria does out of it, or to cause the whole plant to act as veratria does when given alone.

Thirdly, there is nothing recorded by writers as to the actions of veratrum viride, which tends, in any degree, to support the idea that it acts as an intestinal irritant, or as a purgative; and that it is capable of causing, or likely to cause, as veratrum album undoubtedly will, intestinal inflammation, when given in excessive doses.

Hence, lastly, we are unwilling to accept the allegation, even backed up by the verdict of an intelligent jury of Niagara county, that twenty drops of Thayer's fluid extract of this plant, in two doses, was the harmful cause of a two weeks' dysentery and a year's impaired health.

The expert testimony in the case was substantially as regards the actions of the plant, as above given. Perhaps the jury thought the Doctors had combined to tell tough stories, and support each other in statements hardly "on the verge" of truth. If so, their ignorance misplaced the toughness of the stories. It is certainly rather mortifying that the testimony of half a dozen physicians of good repute, should apparently have not the slightest weight; especially upon the actions of a well known drug.

